# Intermediate role of gut microbiota in vitamin B nutrition and its influences on human health

**DOI:** 10.3389/fnut.2022.1031502

**Published:** 2022-12-13

**Authors:** Zhijie Wan, Jianheng Zheng, Zhigang Zhu, Lan Sang, Jinwei Zhu, Shizheng Luo, Yixin Zhao, Ruirui Wang, Yicui Zhang, Kun Hao, Liang Chen, Jun Du, Juntao Kan, Hua He

**Affiliations:** ^1^Center of Drug Metabolism and Pharmacokinetics, China Pharmaceutical University, Nanjing, China; ^2^Nutrilite Health Institute, Shanghai, China

**Keywords:** vitamin B, gut microbiota, interaction, absorption, human health

## Abstract

Vitamin B consists of a group of water-soluble micronutrients that are mainly derived from the daily diet. They serve as cofactors, mediating multiple metabolic pathways in humans. As an integrated part of human health, gut microbiota could produce, consume, and even compete for vitamin B with the host. The interplay between gut microbiota and the host might be a crucial factor affecting the absorbing processes of vitamin B. On the other hand, vitamin B supplementation or deficiency might impact the growth of specific bacteria, resulting in changes in the composition and function of gut microbiota. Together, the interplay between vitamin B and gut microbiota might systemically contribute to human health. In this review, we summarized the interactions between vitamin B and gut microbiota and tried to reveal the underlying mechanism so that we can have a better understanding of its role in human health.

## Introduction

Vitamin B is a group of water-soluble essential nutrients that serve as precursors of essential cofactors in numerous metabolic pathways. Vitamin B deficiency would cause a series of diseases including cognitive dysfunction, neuropathy, cardiovascular disease (CVD), and osteoporosis (1–7). Humans cannot synthesize these vitamins *de novo*, so vitamin B is mainly supplied through diet and, to a lesser extent, synthesized by gut microbiota. With the assistance of specific transporters, dietary vitamin B is primarily absorbed in the small intestine while the vitamin B synthesized by gut microbiota is absorbed in the colon, where microbes are densely populated.

As the most dominant member of the gut microbiota, gut bacteria could interfere with the utilization of vitamin B either directly or indirectly (8). Gut bacteria that are capable of synthesizing and supplying an excessive amount of vitamin B for the host, as well as other intestinal microbes are defined as vitamin B-producers. On the other hand, bacteria that can’t produce vitamin B but require vitamin B to maintain normal physiological functions are called vitamin B-consumers. The balance between vitamin B-producers and -consumers determines the role of gut microbiota, whether as suppliers or competitors, to the host. Gut microbiota could indirectly affect vitamin B utilization by interfering with the nutrient absorption process, which is composed of the release of vitamin B from food and its transport across the intestinal epithelial layer (9). Several physiological factors jointly determine the absorption process of vitamin B: digestive enzymes, gut motility, acidity of gastrointestinal tract, the transporters, and the bound proteins. These factors are largely affected by disease progress of inflammatory bowel diseases (IBD), which could be triggered by invasion of the adherent pathogenic microbiota (10–12). In contrast, probiotics could prevent or alleviate IBD, resulting in a normalized physiological feature of the gut (13, 14). Hence, gut microbiota’s bidirectional effects on gut might influence the absorption process of vitamin B.

Moreover, vitamin B supplementation could change the profiles of gut microbiota, including the diversity, abundance, and function. Considering the crucial role of gut microbiota on human health, disruptions of gut microbiota are associated with multiple disease progressions, such as neurological disorders, CVD, obesity, metabolic diseases, and non-alcoholic liver disease (15–22). Since many bacterial functions originate from their metabolites, a variation in the production of metabolites caused by vitamin B could modulate host health. For now, short-chain fatty acids (SCFAs) are the most well-studied small-molecule metabolites of gut microbiota linking vitamin B nutrition to the maintenance of the host’s intestinal homeostasis and benefits of extra-intestinal organs (23). Other metabolites, however, due to limited published knowledge concerning vitamin B’s influences on them, would not be discussed in the current review.

The potential interaction between vitamin B and gut microbiota has raised the interest to study the relationship between vitamin B and gut microbiota. Recently, excellent review papers have been published to reveal the role of vitamin B on gut microbiota, how gut microbiota affect the absorption of vitamin B remains unclear (24, 25). To establish the relationship between vitamin B and gut microbiota, it is required to understand not only the role of gut microbiota on vitamin B absorption, but also the influence of gut microbiota on vitamin B absorption. The purpose of this review is to summarize the role gut microbiota on vitamin B absorption and the indirectly beneficial role of vitamin B on human health *via* gut microbiota.

## Vitamin B1

### Vitamin B1 and human health

Vitamin B1, also known as thiamine monochloride, thiamine chloride, and aneurine, is a thermal unstable water-soluble essential vitamin. Thiamine is essential for all organisms to metabolize carbohydrates and branched-chain amino acids through glycolysis and the tricarboxylic acid cycle (26). Dietary vitamin B1 occurs to be thiamin pyrophosphate (TPP), which is catalyzed by thiamine pyrophosphate kinase in the presence of ATP to produce thiamine. Vitamin B1 performs as a cofactor of transketolase in the pentose phosphate pathway and pyruvate dehydrogenase and α-ketoglutarate dehydrogenase in the mitochondria (27). The World Health Organization/Food and Agriculture Organization recommends a daily intake of 1.1–1.2 mg of vitamin B1 for adults (28). Vitamin B1 deficiency can induce drowsiness, beriberi, polyneuritis, and Wernicke–Korsakoff syndrome (WKS). Thiamine administration reduces the progression of neurological deficits caused by WKS (29). Nutritional intervention for the treatment of hepatic encephalopathy includes adequate, but not excessive, vitamin B1 supplementation (30).

### Dietary vitamin B1 release and absorption

**FIGURE 1:**
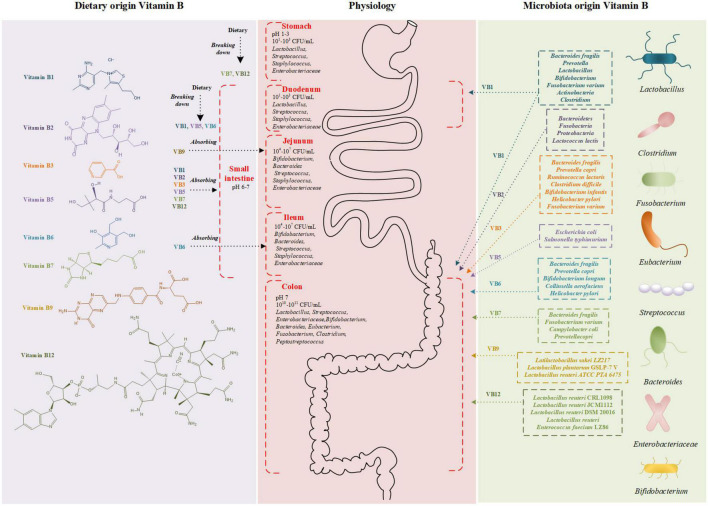
Schematic diagram of vitamin B structure and absorption sites. Vitamin B derived from diet **(left)** and microbiota **(right)** are absorbed in gastrointestinal tract **(middle)**. The different colors represent bacteria producing different vitamin B. CFU, colony forming unit.

Dietary vitamin B1 is primarily absorbed in the small intestine, while those vitamin B1 produced by intestinal microbes are mainly absorbed in the large intestine (Figure 1; 27). Human beings are not able to store free thiamine, and only small amounts of phosphorylated thiamine exist in cells. Therefore, a continuous supply of dietary vitamin B1 is required. Most dietary thiamine is present in phosphorylated forms, which are subsequently hydrolyzed by intestinal alkaline phosphatase to free thiamine. Studies have suggested that the proximal small intestine is the primary site for the absorption of free thiamine (31). Thiamine absorption relies on both unsaturated passive diffusion and saturated active transport (31, 32). In the case of an oral dose below 5 mg, free thiamine is mainly absorbed by the intestinal epithelium through thiamine transporters (THTR-1 and THTR-2, encoded by genes *SLC19A2* and *SLC19A3*) (33). Similarly, microbiota-produced vitamin B1 is absorbed either identically with to the dietary origin or directly by the colon through the TPP transporter (encoding gene *SLC44A4*) (34).

### Influence of gut microbiota on vitamin B1 utilization

Bacteroidetes and Fusobacteria are the most common two bacterial phyla that are able to synthesize TPP (35). Some intestinal microbiota can produce vitamin B1, such as *Bacteroides fragilis*, *Prevotella*, *Fusobacterium varium*, Actinobacteria, and *Clostridium* (35–39). About half number of enzymes presented in these microbiotas take part in the *de novo* synthesis of thiamine (40). However, there also remains gut microbiota in which vitamin B1 synthesis pathway is absent and external source is required for their growth, such as Ruminococcaceae (41). For these bacteria, competing with the host for vitamin B1 is a potential approach for survival, especially in the small intestine.

The gut microbiota may have impact on gastrointestinal functions, therefore affecting the absorption of vitamin B1. *Clostridium botulinum* isolated from the feces of botulism infant can produce thiaminase I to degrade vitamin B1 (42). According to *in vitro* study on Caco-2 cells, thiamin uptake was significantly reduced by Enterotoxigenic *Escherichia coli* infection, which reduces the expression levels of THTR-1 and THTR-2 (43, 44). Transcellular H^+^/OH^–^ gradient has been proved to be the driver of THTR-1 and THTR-2 dependent thiamine transport (33). In addition, vitamin B1 is easy to be oxidized and lose activity in neutral and especially alkaline environment. Lactic acid bacteria can produce acids such as *Bifidobacterium*, *Lactobacillus*, *Enterococcus*, and *Streptococcus* in the intestinal tract (45, 46). The growth of these microbiota is then deduced to improve the absorption of thiamine *via* their regulation of intestinal pH. In the study of Ibrahim Elmadfa et al., the probiotic yogurt microbiota including *Lactobacillus casei* GG could reduce vitamin B1 bioavailability in humans (47). In contrast, yogurt containing *Lactobacillus.* exhibited an oppositive effect on plasma vitamin B1 levels in healthy adult humans, and this observation might be attributable to the complex interactions among vitamin B1, gut microbiota, and host (48). Thus, further studies are required to reveal these complex interactions.

### Indirect role of vitamin B1 on human health as mediated by gut microbiota

Vitamin B1 is essential to the growth of microorganisms, therefore changing the form of gut microbiota. In the absence of thiamine, the population of thiamine acquisition mutant strain was rapidly decreased *in vitro* (40). An initial population of 90% had fallen to 0.1% by day 5. Another study found that the *Eubacterium rectale* A1-86, *Roseburia intestinalis* M50/1 were unable to grow in the absence of thiamine even though they have the genes encoding thiamin synthesis (39). By contrast, the growth level was improved when supplied with thiamine. With the increase of exogenous thiamine, the population of thiamine acquisition mutant was increased *in vitro* (40). The optimal growth was achieved in human intestinal thiamine-nutrient-deficient bacteria, such as *Subdoligranulum variabile* DSM 15176, *E. rectale* A1-86, *R. intestinalis* M50/1, and *Roseburia inulinivorans* A2-194, when thiamine concentrations were at or above 5 ng/mL (39). The relative abundance is correlated with some intestinal bacteria that require peripheral intake of vitamin B1. A positive correlation was reported between vitamin B1 and the relative abundance of Ruminococcaceae (Firmicutes phylum), which lacks the vitamin B1 synthesis pathway and requires a supply of vitamin B1 from the host diet (41). Supplying thiamine to cows’ diet could increase the abundance of cellulolytic microbiota such as *Bacteroides*, *Ruminococcus* 1, *Pyramidobacter*, *Succinivibrio*, and *Ruminobacter*, and enhance ruminal acetic acid production. Their increase enhanced the fiber degradation and ruminal acetate production, thus improving ruminal function (49).

Vitamin B1 has an important influence on SCFAs production by gut microbiota. *Faecalibacterium*, the most abundant of the *rumen cocci* family, expresses pyruvate iron oxidoreductase to convert pyruvate to acetyl coenzyme in the butyrate production pathway. During this process, vitamin B1 is required as a coenzyme, emphasizing its importance in butyrate production (41).

## Vitamin B2

### Vitamin B2 and human health

As the precursor of flavin mononucleotide (FMN) and flavin adenine dinucleotide (FAD), vitamin B2 (Riboflavin) plays a crucial part in multiple biological redox reactions, energy metabolism, antioxidant, and anti-inflammatory, synthesis, and activation of vitamin B6 and vitamin B9 (50–52). Considering the protective role of vitamin B2 in various medical conditions, vitamin B2 deficiency is related to multi-system dysfunction. Lack of vitamin B2 can alter cellular metabolic homeostasis, which leads to night blindness, cataracts, anemia, migraines, and dermatological symptoms (53, 54). Early diagnosis coupled with riboflavin supplementation can heal up ariboflavinosis in an early stage. In addition, a previous study showed that vitamin B2 intake was inversely associated with colorectal cancer and its adequate supply can effectively lower blood pressure in hypertensive patients identified with the *MTHFR 677TT* genotype (55, 56).

### Dietary vitamin B2 release and absorption

Dairy products, green vegetables, fruits, eggs, and meat are excellent sources of vitamin B2 (57). The absorption of vitamin B2 depends on the riboflavin hydrolyzed from FMN and FAD by alkaline phosphatases and FMN/FAD pyrophosphatases (Figure 1; 58, 59). Enterocytes actively involves in the absorption of vitamin B2 through a carrier-mediated process, contributing up to 30 mg of riboflavin at each meal (57). Following cellular uptake, riboflavin may generate FMN by ATP-dependent phosphorylation, and most FMN is further converted to FAD. Vitamin B2 can be absorbed through the basolateral membrane of the enterocytes in the form of free riboflavin or FMN, and then released into the portal blood toward the liver (60).

### Influence of gut microbiota on vitamin B2 utilization

*De novo* synthesis pathway of vitamin B2 was found in nearly all genomes of Bacteroidetes, Fusobacteria, and *Proteobacteria*. It is estimated that 65% of bacterial genomes (a total number of 256 genomes), can produce vitamin B2 according to a systematic genome assessment of B-vitamin biosynthesis (35). Interestingly, *Clostridium acetobutylicum*, *Eremothecium ashbyii*, *Ashbya gossypii*, *Bacillus subtilis*, and many other strains have been industrialized to produce vitamin B2 (61). Vitamin B2 produced by gut microbiota is mainly adsorbed in the colon, serving as an additional source of daily vitamin B2 uptake other than dietary supply (59). Over-expression of riboflavin biosynthesis genes in *Lactococcus lactis* could switch its role from a consumer into a producer of vitamin B2. Compared with the native strain, administration of *L. lactis* modified to produce riboflavin in riboflavin-deficiency rats could improve their riboflavin status, suggesting its importance on maintaining the state of vitamin B2 (62).

Gut microbiota may change the physiological or pathological conditions of the gastrointestinal tract, and then influence the absorption of vitamin B2. The decreased gastric emptying rate has been reported to improve the bioavailability of vitamin B2 (63). *Lactobacillus reuteri* and *Lactobacillus gasseri* OLL2716were able to slow down the gastric emptying rate. The phenomenon indicated that these species of bacterial have a potential influence on vitamin B2 absorption (64, 65). On the other hand, several bacteria could intervene the gastric disease progression. For example, a lower plasma level of vitamin B2 was observed in gastric cancer patients with the presence of *Helicobater pylori* than in patients without infections (66). Meanwhile, lactic acid bacteria, as complementary treatments for intestinal inflammation, have been proved to be effective in the suppression or prevention of *Helicobater pylori* infection (67). These results indicate that gut microbiota may affect the absorption of vitamin B2 by the way of changing rate of gastric emptying (48).

### Indirect role of vitamin B2 on human health as mediated by gut microbiota

The growth of anaerobic bacteria *Blautia coccoides*, *R. intestinalis* and *Enterococcus faecalis* was stimulated by vitamin B2 *in vitro* (68). Replenishment of vitamin B2 has been reported to alter the composition and β diversity of gut microbiota in mice (69). Colon-targeted vitamin B2 supplementation for 3 weeks improved the α diversity of gut microbiota in healthy volunteers but not in Crohn’s disease patients, suggesting that effects of vitamin B2 on gut microbiota are different between groups of people with different physiology situation microbiota (70–72). In clinical interventions, vitamin B2 supplementation for 2 weeks increased the relative abundance of *F. prausnitzii* in healthy individuals (73). Moreover, treatment of vitamin B2 and vitamin C significantly reduced the number of *Proteobacteria*, and showed a trend to increase the number of Firmicutes and decrease the number of Bacteroidetes (70). Another study also reported that the relative abundance of *Streptococcus* was negatively correlated with dietary intake of vitamin B2 (74). Several groups of *Streptococcus* are thought to be correlated with strep infection, such as *Streptococcus pyogenes* and *Streptococcus pneumoniae*. Consequently, sufficient daily intake of vitamin B2 might protect the host from strep infections. In volunteers with migraine, daily supplementation of vitamin B2 increased the abundance of anaerobic bacteria *Roseburia*, and *F. prausnitzii*, while decreased the abundance of *E. coli* (75).

Vitamin B2 forms part of the electron transfer flavoprotein complex of butyryl-CoA dehydrogenase. Consequently, butyrate production is directly affected by vitamin B2 (39). Concentration of SCFAs produced by human gut isolates rely upon vitamin B2 in *in vitro* condition (39). Furthermore, cecal SCFAs content of vitamin B2 depletion-repletion mice model was remarkably increased by the repletion of vitamin B2 (69). *Faecalibacterium prausnitzii* is an effective producer of SCFA, especially butyrate, and a 2-week vitamin B2 supplementation in healthy individuals could increase the abundance of *Facealibacterium. prausnitzii*, which is an effective producer of SCFAs, especially butyrate (39, 75, 76). A randomized trial showed that butyrate production was significantly increased in groups treated with 50 and 100 mg/d of vitamin B2 (73).

## Vitamin B3

### Vitamin B3 and human health

Nicotinamide (NAM) and nicotinic acid (NA) are collectively referred to as vitamin B3 (77). The functional cofactors derived from vitamin B3 are nicotinamide adenine dinucleotide and nicotinamide adenine dinucleotide phosphate, providing reducing equivalents for cellular biochemistry and energy metabolism (78, 79). Vitamin B3 is considered as a potent antioxidant capable of protecting the brain’s cellular membranes and is deemed to be effective in neurodegenerative diseases (80). Aging-related nicotinamide adenine dinucleotide deficiency in the retina is considered as a possible reason for glaucoma in the elderly, and oral administration of vitamin B3 could alleviate the symptoms (81). The most typical disease caused by vitamin B3 deficiency is pellagra, which is characterized by inflammation of mucous membranes, skin lesions, diarrhea, and dementia. Besides, NA is widely used to treat lipid disorders (82).

### Dietary vitamin B3 release and absorption

Vitamin B3 can be obtained from both endogenous and exogenous sources. The endogenous source is produced from tryptophan, and the exogenous source is dietary NA and NAM (Figure 1) (83). NAM mainly exists in animal products, while NA mainly exists in plant-based foods such as beans. With human-derived intestinal epithelial Caco-2 cells and purified isolated brush-border membrane vesicles, a study on the mechanism of NA uptake in humans suggested the existence of an acidic pH-dependent and carrier-mediated mechanism in the physiological range of niacin concentrations (84). Based on the environmental acidity, NA is absorbed in the stomach and the upper part of small intestine by proton co-transporters and an anion antiporter (84–86). Besides, it has also been reported that vitamin B3 could be absorbed by passive diffusion at higher concentrations (87).

### Influence of gut microbiota on vitamin B3 utilization

Several gut microbial species possess vitamin B3 biosynthesis pathways and can synthesize vitamin B3 from tryptophan (83). Bacteria with vitamin B3 biosynthesis pathway include *B. fragilis*, *Prevotella copri*, *Ruminocccus lactaris*, *Clostridium difficile*, *Bifidobacterium infantis*, *H. pylori*, and *F. varium* (35, 88). Nabokina et al. reported that NA uptake by Caco-2 cells increased when the extracellular pH reduced from 8.0 to 5.0 (84). Considering that the absorption of vitamin B3 is pH-dependent, we can deduce that lactic acid bacteria, such as *Bifidobacterium*, *Lactobacillus*, *Enterococcus*, and *Streptococcus*, can change the intestinal acidity and influence the absorption of NA.

### Indirect role of vitamin B3 on human health as mediated by gut microbiota

For now, the role of vitamin B3 on gut microbiota remains unclear. Fangmann et al. found a reduced α diversity and a decreased abundance of Bacteroidetes in the microbiome of obese subjects under a low dietary NA intake (89). For this reason, delayed-release microcapsules targeting the ileocolonic region were developed to deliver vitamin B3 to the microbiome, avoiding the adverse side effects of NA. Such vitamin B3 containing microcapsules significantly increased the abundance of *Bacteroidete*s in human gut (89). Another study has also shown that NA plays an essential role in maintaining normal intestinal homeostasis, reducing intestinal inflammation, and regulating the production level of intestinal antimicrobial peptides (90). The supplementation of NA was also reported to increase the production of acetate while reducing the ratios of propionate/acetate and butyrate/acetate in the colonic contents of piglets, suggesting a beneficial effect of NA on SCFAs production (91).

## Vitamin B5

### Vitamin B5 and human health

Vitamin B5, also known as pantothenic acid, is available in a variety of plants and animal products and unprocessed grains (92). Food-derived vitamin B5 is converted to pantethine and then to Acetyl CoA and acyl carrier protein (93). These two compounds are critical in burning fats and carbohydrates in energy metabolism. Acetyl CoA is also required to produce adrenal hormones including Cortisol (steroid hormone). Hence, vitamin B5 deficiency is associated with impaired adrenal cortical function (94). Patients with rheumatoid arthritis were observed with lower blood pantothenic acid and the severity of arthritis is negatively correlated to the level of vitamin B5 (95). The most common symptoms associated with vitamin B5 deficiency were headache, fatigue, and a sensation of weakness. Health volunteers fed a semisynthetic, pantothenic acid-free diet for 9 weeks developed subclinical signs of fatigue ad listlessness without clinical symptoms (96). Low serum vitamin B5 level also associates with an increased incidence of hypertension (97).

### Dietary vitamin B5 release and absorption

The intestinal tract is exposed to two sources of vitamin B5: diet- and bacteria-origin (98). Dietary vitamin B5 exists mainly in the form of coenzyme A, which is hydrolyzed to pantetheine by alkaline phosphatase and then quickly converted into the absorbable forms of pantothenic acid by pantetheinase in the intestinal lumen (Figure 1; 99). At low luminal concentrations, free pantothenic acid is actively transported *via* the sodium-dependent multivitamin transporter (SMVT, *SLC5A6*) (100–103). At higher concentrations, passive diffusion of vitamin B5 occurs, and no significant difference in the transport rate in different segments of the intestine. It is indicated that absorption of the bacterially synthesized vitamin B5 in the large intestine also involves the SMVT system, though direct evidence is lacking (100, 103, 104).

### Influence of gut microbiota on vitamin B5 utilization

Vitamin B5-producing bacteria and vitamin B5-consuming bacteria coexist in the intestinal tract (25). Vitamin B5-producing bacteria include *E. coli* and *Salmonella typhimurium. Escherichia coli* can utilize aspartate and intermediate metabolites of valine biosynthesis as substrate (24, 105–107). *Salmonella typhimurium* produces pantothenate from alpha-ketoisovalerate using acetohydroxy acid synthase isozyme I and dihydroxy acid dehydratase enzymes (108). Other bacteria, such as *Lactobacillus helveticus*, *Streptococcus*, and *E. faecalis*, members of the vitamin B5-non-producing Firmicutes phylum, require vitamin B5 for their growth *in vitro*, which might compete vitamin B5 with the host (109, 110).

For now, the regulatory effect of gut microbiota on vitamin B5 absorption is still unclear. However, extensive evidence supports that utilization of bacterially synthesized vitamin B5 depends on the gut microbiota, especially in vitamin B5 deficiency. Chemicals containing sulfonamide functional group, including succinylsulfathiazole and sulfathiazole, can change the composition and function of the gut microbiota (111, 112). It was reported that mice consuming the vitamin B5-deficient diet and receiving succinylsulfathiazole exhibit signs of pantothenic acid deficiency (111). A similar phenomenon has also been observed in rats (112). Moreover, the addition of succinylsulfathiazole to a vitamin B5-normal diet deteriorated the deficiency of pantothenic acid, resulting in signs of pantothenic acid deficiency characterized by achromotrichia and porphyrin-caked whiskers appearing in 3 weeks.

### Indirect role of vitamin B5 on human health as mediated by gut microbiota

At present, little is known about the role of dietary vitamin B5 supplementation on gut microbiota. Enhanced vitamin B5 intake appears to increase the relative abundance of *Prevotella* and Actinobacteria and to decrease the abundance of *Bacteroides* in lactating women (113). Non-linear effects of vitamin B5 on the diversity and abundance of intestinal microbiota were observed. In fish, a diet supplemented with 26.0 mg/kg of vitamin B5 increased the diversity and abundance of intestinal microbiota compared with other levels of vitamin B5 supplementation (114). The maximum portion of *Proteobacteria*, Firmicutes, and Mycoplasma appeared in the 20.0 mg/kg, 26.0 mg/kg, and the no-vitamin B5-supplement group, respectively. The minimum portion of *Proteobacteria*, Firmicutes, and Tenericutes appeared in the 37.0, 20.0, and 26.0 mg/kg groups. Among the investigated bacterial species, Mycoplasma is suggested to be correlated with infectious disease pathogenesis (115, 116). It can be inferred from the experimental results that vitamin B5-supplement could inhibit the number of Mycoplasma thus increasing the anti-infection abilities. An *in vitro* study of *L. helveticus* demonstrated that a vitamin B5-deficient medium greatly inhibited the synthesis of fatty acid and protein, and this observation is likely to be explained by the downregulated expression of genes associated with fatty acid synthesis and biotin metabolism (117). Together, these results indicate that deficiency of vitamin B5 might change the growth profile and the biological function of intestinal microbiota.

## Vitamin B6

### Vitamin B6 and human health

Vitamin B6 is a group of compounds with a defined structure, including pyridoxine (PN), pyridoxal, and pyridoxamine, and all of them have 5-position phosphoryl derivatives (118). All these compounds cannot be synthesized by the human body and are mainly provided by diet (119). Animal products mostly contain pyridoxal 5’-phosphate (PLP) and pyridoxamine 5’-phosphate, while pyridoxine 5’-phosphate (PNP) is the dominant form in plant products (119–122). The recommended daily intake amount is 1.3∼1.7 mg for adults (123). Vitamin B6 is a cofactor in many metabolic reactions that include amino acid metabolism, biosynthesis, and degradation of sphingolipid and carbohydrate metabolism (120, 124, 125). Moreover, PLP is the primary active form among all forms of these above derivatives. It acts as a cofactor for many enzymes, such as tryptophan synthase (126), *O*-acetylserine sulfhydrylase (127), 5-Aminolevulinate synthase (128), serine hydroxymethyltransferase (129), and branched-chain amino acid aminotransferase (130). Vitamin B6 deficiency may cause neuromuscular irritability, peripheral neuropathy, dermatitis, stomatitis, cheilosis, depression of the immune system, and sideroblastic anemia (131). Moreover, vitamin B6 has also shown its importance in maintaining a regular function of cognition and keeping the elderly away from CVD (132, 133). Additionally, supplementation of vitamin B6 could protect against reactive oxygen free radicals (132, 134, 135).

### Dietary vitamin B6 release and absorption

The phosphorylated form of vitamin B6 in the diet is hydrolyzed by pyridoxal phosphatase and then absorbed in the intestinal lumen (Figure 1; 136). Many studies demonstrate that, under normal circumstances, the concentration of vitamin B6 in food is not saturable and vitamin B6 enters intestinal cells by passive diffusion (131). However, a study with Caco-2 cells has recently challenged this concept. It gives clear evidence for a specialized, carrier-mediated system for the uptake of PN (120). Another study finds that the unique carrier-mediated mechanism for PN uptake functional exists in mammalian colonocytes (124). And these results indicate that the uptake of vitamin B6 could be affected by several extracellular and intracellular factors (124). As an instance, Said et al. demonstrated a pH-dependent and amiloride-sensitive system involving PN uptake in intestinal epithelial cells (120).

### Influence of gut microbiota on vitamin B6 utilization

For humans, there are two primary sources of vitamin B6, one is from the diet, and the other is produced by normal gut microbiota (124). For example, *B. fragilis* and *P. copri* (Bacteroidetes), *Bifidobacterium longum* and *Collinsella aerofaciens* (Actinobacteria) and *H. pylori* (*Proteobacteria*), and these species of bacteria can produce vitamin B6 (35). Furthermore, many studies have shown that there are two biosynthetic pathways, the deoxyxylulose 5-phosphate (DPX)-dependent pathway for PLP and the DPX-independent pathways for PNP (137), and different gut microflora decide on different pathways to choose (35).

Under normal circumstances, dietary and bacterial sources can offer enough vitamin B6 for the human body to absorb, and deficiency of vitamin B6 is a rare phenomenon (114). However, some researchers reported that drugs, alcohol, and smoking, deoxyxylulose 5-phosphate (DPX) (138). Furthermore, these factors above could change gastrointestinal motility, which might bring about a narrowed absorption window and a reduced bioavailability for vitamin B6 (139, 140). Moreover, such intestinal environmental factors could change gut microbiota composition (141–144). Ferrer et al. found that gut microbiota in the lean gut seems to get more involved with vitamin B6 biosynthesis and offer more vitamin B6 for absorption (145, 146). At the same time, the hydrolysis of vitamin B6 is a significant procedure for absorbing vitamin B6 and is closely related to the pH of the intestinal lumen. Since some species of lactic acid bacteria, including *Bifidobacterium, Lactobacillus*, *Enterococcus*, and *Streptococcus* (45–48), can produce acid to lower the pH, we may infer that variation in the proportion of these specific bacterial can influence vitamin B6. Meanwhile, alkaline phosphatase is the essential enzyme during this process. It is also able to affect the growth of gut microbiota (147). The intervention between alkaline phosphatase and gut microbiota is complicated, and it is essential to figure out its influence on vitamin B6 absorption. Unfortunately, there is not much investigation about it.

### Indirect role of vitamin B6 on human health as mediated by gut microbiota

In the intestine, vitamin B6 serves as essential nutrients for the gut microbiota (25). Some species of microbiota lack the ability to biosynthesize vitamin B6, such as most genera within the Firmicutes phylum (*Veillonella*, *Ruminococcus*, *Faecalibacterium*, and *Lactobacillus* spp.) (35). Hence, they acquire exogenous vitamin B6 from the intestinal tract to maintain their life activities. The proportion of food compositing vitamin B6 can influence the profile of the gut microbiota. For example, a larger amount of vitamin B6 absorbed from food was associated with greater richness and evenness of the gut microbiota (124). Mayengbam et al. investigate the composition of gut microbiota and their metabolites in a rat model with vitamin B6 deficiency. They reported that arginine biosynthesis was impaired in this rat model, and the vitamin B6 metabolism was affected. Arginine not only performs as a substrate for protein synthesis but also as a precursor for a variety of molecules linked to cell function, such as nitric oxide. Insufficient vitamin B6 might interferes the host’s *de novo* protein synthesis and related cell functions. Vitamin B6 produced by microbiota is not enough for gut microbiota, and the composition of gut microbiota is changed for this reason (148).

## Vitamin B7

### Vitamin B7 and human health

Vitamin B7, also known as biotin, acts as a cofactor for multiple carboxylases that are associated with fatty acid, glucose, and amino acid metabolism (149). Vitamin B7 is exclusively synthesized by plants and microbiota such as bacteria and yeast, so vitamin B7 synthesized by microbiota in human large intestine make an important contribution to daily supplements for human in addition to food. The adequate daily requirement of vitamin B7 is 35 μg for infants and 150–300 μg for adults. And it is relatively non-toxic even at doses of greater than 60 mg/day for several months (149). Vitamin B7 participates in normal immune function, maintaining the integrity of the intestinal mucosa or homeostasis. It also plays an important role in maintaining skin health and anti-inflammation *via* inhibiting NF-κB activation (101, 150). Therefore, severe vitamin B7 deficiency results in skin abnormalities, neurological disturbances, and growth retardation. Symptoms of vitamin B7 deficiency contain inflammation, loss of appetite, glossitis, dandruff dermatitis, and hair removal. Hence, therapeutical vitamin B7 supplementation can improve hair loss and prevent not only seborrheic hair loss, but also juvenile gray hair in the pathological case of biotin deficiency (151). Although neuroleptic effect of vitamin B7 has not been proven, it does demonstrate a beneficial effect on treatment of depression and insomnia (139).

### Dietary vitamin B7 release and absorption

Humans are exposed to two sources of vitamin B7, including dietary sources and bacterial source in the large intestine (9). Dietary vitamin B7 exists in either free form or protein-bound form. Ingested protein-bound forms of vitamin B7 are firstly broken down by gastrointestinal proteases and peptidases to biocytin (biotinyl-L-lysine) and biotin-oligopeptides (Figure 1). These products are further processed in the intestinal lumen to release free biotin before absorption. Free biotin from the gastrointestinal tract is rapidly absorbed. Absorption of free biotin by the proximal intestine is mediated by SMVT, which also transports vitamin B5 and antioxidant lipoates (136, 142). In the intestine, SMVT is exclusively expressed on the apical membrane of polarized intestinal absorptive cells and thus SMVT system is the only biotin uptake system in the mammalian gut (104).

### Influence of gut microbiota on vitamin B7 utilization

It has long been recognized that the normal microbiota of large intestine can synthesize large amounts of biotin. Vitamin B7-producing microbiota includes *B. fragilis*, *F. varium*, and *Campylobacter coli* (24). Meanwhile, vitamin B7-consuming bacteria must gain vitamin B7 from the environment to maintain microbial functions and these bacteria lack the vitamin B7 biosynthetic pathways. For instance, *Lactobacillus* possesses genes involved in obtaining biotin from environments (145).

Human body lacks the capacity to produce vitamin B7, for which vitamin B7 is mainly supplied by the jejuna and to a lesser extent, from the distal gut. However, absence of gut microbiota may negatively affect circulating vitamin B7 levels. In rodent model, enhanced vitamin B7 transport was observed in decreased intestinal pH (152). Since lactic acid bacteria such as *Bifidobacterium*, *Lactobacillus*, *Enterococcus*, and *Streptococcus* can produce lactic acid and lower the local acidity in the intestinal lumen (45, 46), it is suggested that supplementation of lactic acid bacteria might increase the absorption of vitamin B7. Rat intestinal infection of *Salmonella enterica* serotype *Salmonella typhi* results in a significant reduction in intestinal vitamin B7 intake (150). Obese mice induced by a high-fat diet demonstrate a changed gut microbiota profile, resulting in fewer microbes expressing genes for vitamin B7 synthesis (153). As a result, a reduction in vitamin B7 synthesis and lowered plasma vitamin B7 levels in obese mice were observed, indicating the significant importance of intestinal microbiota in maintaining vitamin B7 levels in obesity (153).

### Indirect role of vitamin B7 on human health as mediated by gut microbiota

The composition of gut microbiota may be influenced by vitamin B7. Vitamin B7-consuming bacteria with free biotin transporter, including *Prevotella*, *Bifidobacteria*, *Ruminococcus*, and *Lactobacillus*, require vitamin B7 to maintain their microbiological functions (154). Hence, vitamin B7 deficiency might interfere with the abundance of the above bacteria. For instance, deprivation of vitamin B7 has been reported to cause intestinal dysregulation and overgrowth of *Lactobacillus murine* (155).

## Vitamin B9

### Vitamin B9 and human health

Vitamin B9 (folate) is a micronutrient for the synthesis and functional regulation of many biomacromolecules in humans (156, 157). In fortified foods, supplements, and pharmaceuticals, vitamin B9 occurs in the synthetic form of folic acid (158). As a critical cofactor in one-carbon metabolism, vitamin B9 could transfer carbon units in methylation reaction, DNA and RNA biosynthesis, and amino acid metabolism (159). Megaloblastic anemia is one of the most common symptoms of vitamin B9 deficiency. The main reason for this disease is the inhibition of the maturation of erythropoietic precursors (159, 160). Lack of vitamin B9 also correlates with neural tube defects (161). Furthermore, vitamin B9 inadequacy is associated with the pathogenesis of several chronic diseases including CVD, cancers (colorectal, prostate, and breast cancer) (162–164), and Alzheimer’s disease (160, 165, 166).

### Dietary vitamin B9 release and absorption

Natural folate and folic acid have similar absorption processes (158). In food, vitamin B9 usually occurs as folate polyglutamate, which is hydrolyzed to the monoglutamate form by glutamate carboxypeptidase II before the absorption in the brush border of the proximal part of the jejunum (Figure 1; 167). Monoglutamate folate can be transported by the proton-coupled folate transporter (PCFT) across the apical membrane of enterocytes (168, 169). After vitamin B9 is metabolized to 5-methyl-tetrahydrofolate in the enterocytes by dihydrofolate reductase, it can be transported by multidrug resistance-associated protein (MRP) into the portal vein. Vitamin B9 could experience enterohepatic circulation, which means it can be discharged into the bile and then reabsorbed in the intestine. Bacteria synthesized folate might be absorbed in the colon because the colon has abundant PCFTs for vitamin B9 absorption (170, 171). Moreover, the enzymes that favor the absorption of vitamin B9, such as folate hydrolase, γ-glutamyl hydrolase, and folate hydrolase 2, are also highly expressed in the colon (172, 173).

### Influence of gut microbiota on vitamin B9 utilization

The gut microbiota also plays a valuable role in producing and consuming vitamin B9 (172, 174). According to an evaluation of human gastrointestinal bacterial genomes, 13.3% of the bacteria possess the ability of vitamin B9 *de novo* synthesis, and 39% could produce vitamin B9 with extra para-aminobenzoic acid provided from other bacteria or food (172). It is also reported that 26% of Actinobacteria, 71% of *Proteobacteria*, 79% of Fusobacteria, and 15% of Firmicutes in the human gut microbes have the potential to *de novo* synthesize vitamin B9 (35). A systematic genome evaluation of the vitamin B family suggested human intestinal microorganism is capable of producing 37% of daily required vitamin B9 in non-pregnant adults (35). Folate-producing strains have been extensively screened to fortify vitamin B9 content (175–177). Liu et al., have isolated *Latilactobcillus sakei* LZ217, a good producer of vitamin B9, from raw milk (176). Zhang et al. screened high vitamin B9-producing strains from 12 lactic acid bacteria and then obtained its variant, *Lactobacillus plantarum* GSLP-7 V after stressing with drugs (177). Based on a vitamin B9 deficient rat model induced by a vitamin B9-free diet, they further proved GSLP-7 V and its fermented yogurt could restore serum vitamin B9 and homocysteine (Hcy) to normal levels. A case that is closer to clinical application is *L. reuteri* ATCC PTA 6475. It has been proved to be safe for humans and could produce vitamin B9 with additional para-aminobenzoic acid (172). Collectively, the microbiome has beneficial potential in treating vitamin B9 deficiency. Another study pointed out that 86% of the 512 investigated bacterial reference genomes required vitamin B9 or its intermediates from food or other microbiota (172).

### Indirect role of vitamin B9 on human health as mediated by gut microbiota

A vitamin B9-supplement diet slightly increased gut bacterial community richness according to the abundance-based coverage estimator, compared to a vitamin B9-deficient diet in the high-fat-diet-induced obesity mouse model. The relative abundance of Actinobacteria was significantly increased, while it is the opposite for *Clostridia* (178). However, Wang et al. proved additional folic acid didn’t make significant differences in the indices of diversity in the cecum, but increased relative abundance of *Lactobacillus salivarius*, *L. reuteri*, and *Lactobacillus mucosae* (179). Vitamin B9 deficiency can influence bacterial diversity. Compared to a micronutrient-sufficient diet in gnotobiotic mice, the vitamin B9 deficiency diet increased β diversity after 21-day treatment. But a 14-day full diet treatment did not change this trend (180). Another study based on human gut microbiota assessed fecal microbiota composition. The fecal microbiota community has lower α and β diversity when healthy volunteers with less vitamin B9 diet. And the fecal microbiota community has a higher potential to produce vitamin B9 *in vitro* experiments (181). It’s suggested that vitamin B9 deficiency probably decreases the richness of human gut microbiota.

Vitamin B9 also can influence the amount of SCFAs in the gastrointestinal tract. In the study of Wang et al., there were more acetic acid and valeric acid in the cecum and colon of weaned piglets fed with vitamin B9 supplementation (179). Liu et al. proved the vitamin B9-produced probiotics, *L. sakei* LZ217, could increase SCFAs content, especially for propionic acid and butyric acid in the fecal slurry cultures (176).

## Vitamin B12

### Vitamin B12 and human health

Vitamin B12 (cobalamin) is a member of the corrinoids that is required by methionine synthase and methylmalonyl-CoA mutase (182, 183). Methionine synthase is pivotal in catalyzing the conversion of Hcy to methionine. The subsequently adenosylated of methionine would generate S-adenosylmethionine to supply methyl groups for biological methylation modifications of proteins and nucleic acid. Methylmalonyl-CoA mutase is involved in mitochondrial metabolism. A daily intake of 4 μg is adequate to maintain the normal biological functions of vitamin B12 (184), which could be satisfied by dietary supplementation with 5–30 μg (185). Vitamin B12 deficiency is correlated with several pathological progressions due to its important role in methylation and catabolism. Without sufficient vitamin B12 to convert total homocysteine (tHcy) to methionine, circulating level of accumulated tHcy might increase the risk of CVD (186, 187). Moreover, vitamin B12 deficiency is responsible for cognitive impairment and neurological disorders, which might result from the accumulation of tHcy and methylmalonic acid (188–190). In addition, deficiency of vitamin B12 has also been reported to perform a positive association with osteoporosis (191), macular degeneration (192, 193), and frailty (194).

### Dietary vitamin B12 release and absorption

For human, the major source of vitamin B12 is animal products while intestinal microbiota synthesis might also contribute to a minor fraction (195). Vitamin B12 could be absorbed by both passive diffusion and receptor mediated endocytosis in the intestine (Figure 1). Passive diffusion is negligible in physiological doses (100–1000 μg) supplied by food or supplementation (196). The absorption of vitamin B12 by receptor mediated endocytosis is a multistep process (197–199). In the upper gastrointestinal tract, vitamin B12 is released from the protein carriers with the assistance of gastric acid and pepsin and then binds to haptocorrin under the acidic condition. After the degradation of haptocorrin by pancreatic proteases, the released vitamin B12 binds to intrinsic factors in duodenum. The generated vitamin B12-intrinsic factor complex is then endocytosed by mucosal cells in the distal ileum with the help of receptor cubilin, transmembrane protein amnionless, and megalin/LRP2. After entering the mucosal cells, vitamin B12-intrinsic factor complex is dissociated from cubilin in the early endosomal compartment. In the lysosome, intrinsic factor is degraded and released vitamin B12 enters the cytoplasm *via* LMBD1. The exit of free vitamin B12 from enterocyte might depend on MRP1. With enterohepatic circulation, the secreted vitamin B12 in duodenum would bind to intrinsic factor and then reabsorbed into the circulation.

### Influence of gut microbiota on vitamin B12 utilization

Intestinal microbiotas are either producers or consumers of vitamin B12. Moreover, the intestinal absorption of vitamin B12 could be in turn influenced by intestinal microbiota (35). Several bacteria have been reported to be vitamin B12 producers, such as *L. reuteri*, and *Enterococcus faecium* (200, 201). It is supposed that vitamin B12-producing bacteria supplementation could improve vitamin B12 utilization in the gastrointestinal tract. Such an assumption has been proved in mice fed with vitamin B12 deficient diets. The supplementation of *L. reuteri* CRL1098, a vitamin B12-producing strain, prevented the signs of vitamin B12 deficiency, suggesting the therapeutical effect of intestinal bacteria in vitamin B12 deficiency (202). However, these beneficial effects might be limited if the bacteria are colonized in the colon (203), where lack of necessary transporters. In order to develop a probiotic treatment for vitamin B12 deficiency, the position of bacterial colonization should be considered. Around 80% of microbiota in the gastrointestinal tract are considered as consumers of vitamin B12 (204). Hence, the overgrowth of bacteria might compete with the exogenous vitamin B12 with their host and then reduce the bioavailability (205). In small intestinal bacterial overgrowth, consumption of vitamin B12 by the increased anaerobesis was considered a major reason for vitamin B12 deficient symptoms (206). Reducing the abundance of vitamin B12-consuming bacteria is of benefit to vitamin B12 deficiency. For instance, daily probiotic treatment of *Lactobacillus* performed a beneficial effect on both bacterial overgrowth and vitamin B12 absorption, suggesting the probiotic treatment might improve the vitamin B12 deficiency *via* inhibiting the vitamin B12-consuming bacteria overgrowth (207, 208).

Besides production or consumption of vitamin B12, intestinal microbiota might indirectly change the bioavailability of vitamin B12 *via* exerting influences on the absorption-related physiological factors. Gastrointestinal diseases associated with reduced acid secretion or enzyme content might interfere with the release of vitamin B12 from food (209) or the translation of vitamin B12 to intrinsic factors (199). Reduced vitamin B12 absorption is also observed in IBD, which is characterized by abnormal gut permeability (198, 199). As a probiotic, Lacidofil treatment significantly improved the gastric acid secretion in *H. pylori*-infected mongolian gerbils, which conduced to the release of vitamin B12 from food (210). Some gut bacteria have also presented the remission effect on IBD, which might improve the absorption of vitamin B12 *via* normalizing the gut permeability (211). Besides, excessive competition between gut microbiota and the host might interfere with the bioavailability of vitamin B12. For instance, *Bacteroides thetaiotaomicron* expresses an essential surface-exposed lipoprotein for vitamin B12 transport named BtuG (212). The higher binding affinity of BtuG could remove vitamin B12 from intrinsic factors and reduce vitamin B12 absorption.

### Indirect role of vitamin B12 on human health as mediated by gut microbiota

Vitamin B12 serves as a critical cofactor of diverse enzymes in human gut microbes for nucleotide synthesis, amino acid metabolism, carbon and nitrogen metabolism, and secondary metabolite synthesis (203, 213). The biosynthesis of vitamin B12 involves about 30 enzyme-mediated steps and only a small fraction of bacteria could produce this vitamin (214). Most gut bacteria utilize vitamin B12 that escapes the absorption in the ileum and reaches the large intestine (215). The competition of vitamin B12 in gut microbiota might influence their growth, colonization, and metabolic processes (5, 204, 216).

*In vitro* study of colonic model suggested that vitamin B12 supplementation may increase α diversity, but the results depend on the form and dose of cobalamin administered (217). In another *in vitro* study, α diversity is reduced after methylcobalamin supplementation but not in cyanocobalamin treatment group (218). In mice, no significant difference in α diversity had been observed after vitamin B12 treatment, even under different doses (182, 219). A study suggested that cyanocobalamin supplementation had an increase in the α diversity and exerted a significant difference in β diversity at the genus level (220). However, some studies were unable to support this conclusion (182, 221). In humans, vitamin B12 intake could promote the increase of α diversity in adults but not in infants or children (72, 113, 222, 223). However, the association between vitamin B12 intake and β diversity was only observed in infants at 6 months of age and in older veterans rather than in other observed groups, including infants at the age of 4 or 5 months, lactating women, and children aged between 2 and 9 years (72, 113, 222, 223).

In the colonic model, cobalamin supplementation would increase the relative abundance of Firmicutes and Bacteroidetes and is opposite to *Proteobacteria* and *Pseudomonas* (217). After 7 days of methylcobalamin supplementation, an increased proportion of *Acinetobacter* and declined a fraction of *Bacteroides*, *Enterobacteriaceae*, and Ruminococcaceae had been observed in another colonic model-based study (218). In the studies of murine, the associations between vitamin B12 supplementation and the relative abundance of bacteria have been also reported (182, 219, 220). The supplementation of vitamin B12 has elevated the fraction of Firmicutes and reduced the proportion of Bacteroidetes. Compared to methylcobalamin, cyanocobalamin treatment resulted in higher levels of Bacteroidetes and *Proteobacteria* and lower levels of Firmicutes in mice. In humans, vitamin B12 intake might increase the proportion of *Proteobacteria* (113) and *Verrucomicrobia* (72) and reduce the abundance of Bacteroidetes (224). However, some clinical studies also suggested that vitamin B12 intake had no influence on bacterial abundance (222, 223, 225–227). These controversial results might be due to the different study designs and participants.

*In vitro* study suggested that the addition of cobalamins increases the generation of SCFAs, especially butyrate and propionic acid (218). Another *in vitro* study indicated that low-dose cyanocobalamin-enriched spinach could increase the generation of butyrate and acetate (228). In mice, a reduction of SCFAs has been observed in dietary vitamin B12 restriction (229). However, the effect of oral vitamin B12 on cecal SCFA was absent in mice with dextran sodium sulfate induced colitis (220).

## Conclusion and prospects

**FIGURE 2:**
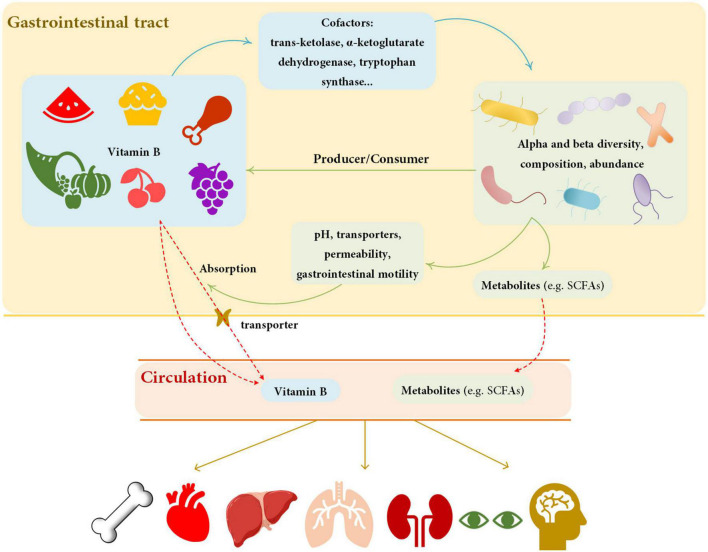
Summary of the relationship between vitamin B and gut microbiota. Vitamin B can affect the diversity, composition, and abundance of gut microbiota by influencing cofactors. Gut microbiota affects vitamin B by producing or consuming and by modifying the physiological properties of the gastrointestinal tract. Solid lines represent effect, dashed lines represent transportation. The blue lines show the effect of vitamin B on gut microbiota, and the green lines show the effect of vitamin B on gut microbiota. Red lines represent the process of substance transfers into circulation. Yellow lines represent the effects of circulation substances on organs. The metabolites of gut microbiota and dietary vitamin B can affect human health. SCFAs, short chain fatty acids.

In this review, we summarized current knowledge about the interaction between gut microbiota and vitamin B nutrition (Figure 2) to infer the consequence of probiotics supplementation, which might be helpful to optimize the treatment of probiotics. Vitamin B perform as essential micronutrients for human. The absorption process of multiple dietary vitamin B requires the assistance of many transporters, which generally occurs in the small intestine (Table 1). At the same time, gut microbiota not only act as producers and/or consumers to modify the supplementation of vitamin B in the gut (Table 2) but also affect the absorption of vitamin B by altering the physiological or pathological factors of the gastrointestinal tract (Table 3). As cofactors of multiple enzymes, supplement with vitamin B can change the diversity, abundance, and functions of gut microbiota (Table 4). Understanding the interactions between vitamin B and gut microbiota can help us prevent vitamin B deficiency and, more importantly, allow us to recognize beneficial potential of probiotics on human healthy.

**TABLE 1:** Summary of vitamin B absorption processes.

Vitamin	Daily intake	Hydrolysis from food	Absorbing location	Transporter
Vitamin B1	1.1–1.2 mg (28)	Intestinal alkaline phosphatase (33).	Small intestine and large intestine (27).	A combination of unsaturated passive diffusion and saturated active transport (31, 32, 230). By intestinal epithelium through THTR-1 and THTR-2 (*SLC19A2* and *SLC19A3*) (33).
Vitamin B2	1.1–1.3 mg (59)	The diet needs to be hydrolyzed to riboflavin through protein denaturation and hydrolysis by alkaline phosphatases and FMN/FAD pyrophosphatases (59, 231).	Small intestine (58, 231).	Specific carrier-mediated processes (57).
Vitamin B3	28 mg for male and 18 mg for female in the diet (79)	Synthesized from tryptophan by pyridine carboxylase (83).	Stomach and upper intestine (84–86).	Proton cotransporters SMCT1 (*SLC5A8*), GPR109A (*HCAR2*) (25).
Vitamin B5	4–7 mg for adults and 5–9 mg for pregnant women (28)	Dietary vitamin B5 is firstly hydrolyzed to pantetheine by alkaline phosphatase in the intestinal lumen, and then is converted into the absorbable forms of pantothenic acid by pantetheinase (99).	Intestinal lumen (99).	At low luminal concentrations, free pantothenic acid is actively transported into the absorptive cells via the SMVT (100–103).
Vitamin B6	1.3–1.7 mg (123)	The vitamin B6 in the diet is hydrolyzed by pyridoxal phosphatase (83, 232, 233).	Jejunum, would also occur in ileum or cecum (234).	
Vitamin B7	150–300 μg for adults, 35 μg for infants (149)	Ingested protein-bound forms of biotin are first broken down by gastrointestinal proteases and peptidases to biocytin (biotinyl-L-lysine) and biotin-oligopeptides (9).	Small intestine (149).	SMVT (100, 136)
Vitamin B9	400 μg for adult, 600 μg for pregnant women (28)	Glutamate carboxypeptidase II, dihydrofolate reductase (167).	Brush border of the proximal part of the jejunum (167).	PCFT (168, 169)
Vitamin B12	5–30 μg (187)	Vitamin B12 is released from the protein carriers with the help of gastric acid and pepsin (197). Pancreatic proteases.	Duodenum (197, 199).	Transmembrane protein amnionless and megalin/LRP2 (197, 199).

**TABLE 2:** Summary of vitamin B-producers and vitamin B-consumers.

Vitamin	Producing bacteria	Consuming bacteria
Vitamin B1	*B. fragilis* (36), *Prevotella* (37), *F. varium* (38), Actinobacteria (39), *Clostridium* (35)	Ruminococcaceae (41), *E. rectale* A1-86 and *R. intestinalis* M50/1 strains (39)
Vitamin B2	The *de novo* synthesis pathway was found in nearly all genomes of Bacteroidetes, Fusobacteria, and *Proteobacteria* (35).	
Vitamin B3	*B. fragilis* and *P. copri* (Bacteroidetes); *R. lactaris*, *C. difficile* (Firmicutes); B. *infantis* (Actinobacteria); *H. pylori* (*Proteobacteria*); and *F. varium* (Fusobacteria) (25).	Bacteroidetes (25)
Vitamin B5	Vitamin B5 producing bacteria include *E. coli* and *S. typhimurium* (24, 105, 106)	*L. helveticus*, *Streptococcus* and *E. faecalis*, members of the vitamin B5 non-producing. Firmicutes phylum, requires vitamin B5 for their growth *in vitro* (109, 110, 235).
Vitamin B6	*B. fragilis*, *P. copri*, *B. longum*, *C. aerofaciens*, *H. pylori* (35)	Most Firmicutes genera (*Veillonella*, *Ruminococcus*, *Faecalibacterium*, and *Lactobacillus* spp.) lack a vitamin B6 biosynthesis pathway (35).
Vitamin B7	Bacteria that can produce vitamin B7 include *B. fragilis*, *F. varium*, and C. *coli* (24).	*Lactobacillus*
Vitamin B9	*L. sakei* LZ217, has shown a good ability of producing vitamin B9 (176). *L. plantarum* GSLP-7 V *L. reuteri* ATCC PTA 6475	Most of them (In 512 bacterial reference genomes, 86% of them required vitamin B9 or intermediates from human food or other bacteria) (172).
Vitamin B12	*L. reuteri* CRL1098 (236), *L. reuteri* JCM1112 (237), L. *reuteri* DSM 20016 (238), *L. reuteri* (201), and *E. faecium* LZ86 (200).	80% of bacteria are predicted as consumers of vitamin B12 in the gastrointestinal tract (204), e.g., *B. thetaiotaomicron*.

**TABLE 3:** Gut microbiota affect the absorption of vitamin B *via* modifying the physiological properties of gastrointestinal tract.

Key physiological factors for nutrient absorption	Influences of microbiota on physiological conditions	Influence on vitamin B absorption
Permeability	↓ the abundance of *Bifidobacterium*, *Faecalibacterium*, and *Lactobacillus* → ↑ gut permeability → ↑ IBD (239, 240) *L. Plantarum*, *L. casei*, *B. infants*, and *S. salivarius* → ↓ gut permeability → ↓ IBD (241–244)	Vitamin B (except vitamin B9) could be absorbed by passive diffusion. Bacterial infection might increase vitamin B amount of absorption.
Gastrointestinal motility	Gut bacteria → SCFAs → ↑ gastrointestinal motility IBD mouse (245–247) *L. casei* and *Bifidobacterium animalis* → SCFAs → ↓ intestinal motility in rats (232, 248–250) Gram-negative bacteria, *E. coli* Nissle and *L. reuteri* →↓ gastrointestinal motility in mice (10, 251–254)	Enhanced gastrointestinal motility resulting from intestinal microbiota might cause a narrowed absorption window (140) and thus results in reduced bioavailability of vitamin B.
The degree of acidity (pH) in gastrointestinal tract	*H. pylori* infection → ↑ pH (255) *Bifidobacterium*, *Lactobacillus*, *Enterococcus*, and *Streptococcus* →↓ pH (45, 46)	The absorption progress of vitamin B1, vitamin B3, vitamin B6, and vitamin B9 are pH-dependent. Lactic acid bacteria might change the rate of vitamin B absorption.
Expression of transporter	*Gordonibacter* → ↓ the expression level and activities of MDR1, BCRP, MRP2, and MRP7 *in vitro* and mice (256, 257) *E. coli* → ↓ the expression of THTR-1 and THTR-2 in a Caco-2 cell model (44) *S. enterica* serovar *Typhimurium* → ↑ CFTR expression in the intestinal epithelium (258), ↓ the transcription of *SLC5A6* *S. typhimurium* → ↑ MRP2 expression in human intestinal biopsy material (259, 260), ↓ transport function of P-gp (260)	Overgrowth of *E. coli* might compromise vitamin B1 absorption due to downregulation of THTR-1 and THTR-2. *S. enterica* serovar *Typhimurium* might reduce absorption of vitamin B5 and vitamin B7 *via* inhibiting the SMVT (100, 150).

**TABLE 4:** Influence of vitamin B on gut microbial profiles.

Name	Diversity	Abundance	SCFAs
Vitamin B1	∼	Ruminococcaceae (Firmicutes phylum) has a positive correlation between the relative abundance and vitamin B1 intake (41).	Involved in the butyrate production pathway (41).
Vitamin B2	Improve microbial α diversity in healthy volunteers (70–72). Change β diversity in mice (69).	*In vitro*, the growth of *B*. coccoides, *R. intestinalis*, and *E. faecalis* was stimulated (68). In humans, increased in *F. prausnitzii*, *Roseburia*, and decrease in *Streptococcus* and *E. coli* (74, 75).	Increased SCFAs content, especially butyrate both in mice and human (39, 69, 70, 75, 76).
Vitamin B3	Improve in obese human subjects (89)	Increase in Bacteroidetes in obese human subjects (89).	Improve SCFAs concentrations in the colon (261).
Vitamin B5	Increased the diversity in Juvenile Golden Pompano (114).	Increases in *Prevotella* and Actinobacteria, decreased in *Bacteroides* in lactating women (113). Increased abundance of intestinal microflora in Juvenile Golden Pompano (114). Vitamin B5 supplements might inhibit the number of *Mycoplasma*.	*In vitro*, fatty acid synthesis and protein synthesis are greatly inhibited without vitamin B5 (117).
Vitamin B6	Positive relationship (124). In rats, vitamin B6 diet showed intestinal microbiota segregation (148).	In mice, a vitamin B6 diet reduced *S. typhimurium* (262).	In vitamin B6-deficient rats, the cecal concentrations of SCFAs (i.e., propionate, butyrate, isobutyrate, valerate, and isovalerate) were decreased, whereas acetate levels were unchanged (148).
Vitamin B7	Improved microbiota diversity in mice (153).	Biotin deficiency causes intestinal dysregulation and overgrowth of *L. murine* (155).	
Vitamin B9	In human, less vitamin B9 in food cause lower α diversity (181). In mice, a vitamin B9 deficiency diet increased β diversity after 21-day treatment (180). Human fecal microbiota (participants with less vitamin B9 diet) had lower β diversity (181).	In weaned piglets, a vitamin B9 diet increase in *L. salivarius*, *L. reuteri*, and *L. mucosae* (179). In obesity mouse model, vitamin B9 diet slightly increased gut bacterial community abundance. The Actinobacteria was increased but opposite for *Clostridia* (178). In human, vitamin B9 deficiency decrease the richness of gut microbiota (178).	In weaned piglets, vitamin B9 diet increase SCFAs content, especially for propionic acid and butyric acid in the fecal slurry cultures, acetic acid, and valeric acid in cecum and colon (176, 179).
Vitamin B12	In colonic model, vitamin B12 increases α diversity (217). In human, vitamin B12 increases α diversity in adults but not in infants or children (72, 113, 222, 223). In mice, cyanocobalamin supplementation exerted significant difference in β diversity at the genus level (220).	In colonic model, methylcobalamin supplementation increased *Acinetobacter* and declined *Bacteroides*, *Enterobacteriaceae*, and Ruminococcaceae (218). In healthy human, vitamin B12 intake might increase the proportion of *Proteobacteria* (113) and *Verrucomicrobia* (72) and reduce the abundance of Bacteroidetes	*In vitro*, cobalamins increases the generation of SCFAs, especially butyrate and propionic acid (218).

In addition to supplements, the probiotic product has recently been approved by FDA as the therapeutical approach for disease treatment, which has significantly extended the application of probiotics. Considering their ability for vitamin B production and intestinal function modification, probiotics might be a potential therapeutical approach for vitamin B deficiency. Although conventional vitamin B supplements have been applied, they are insufficient to treat vitamin B deficiency patients of malabsorption. In this population, improved intestinal absorption rather than vitamin B supplementation would be a more efficient approach to improve vitamin B nutrition. The combination of probiotics regulating intestinal function and vitamin B producers/supplements could further increase the absorption of vitamin B in the gastrointestinal tract. Despite the beneficial effect on human health, risks are remaining in probiotics treatment. The overgrowth of *Lactobacillus murinus* after vancomycin treatment might deplete vitamin B7 available, suggesting the potential risk of nutrition competition after probiotics treatment. For the purpose of reducing the risk of nutrition competition, vitamin B should be supplied along with probiotics. Another consideration of vitamin B supplementations in probiotics treatment is the beneficial effect of vitamin B on the growth and function of gut microbiota. But types and dosages of vitamin B should be optimized as supplements of probiotics treatment. Overall, probiotics treatment could be a potential treatment for vitamin B deficiency, and vitamin B supplement might either reduce the risk of probiotics-induced vitamin B deficiency or improve the efficacy of vitamin B. Nonetheless, these assumptions are still requiring further scientific and clinical studies to optimize the combination of vitamin B and probiotics treatment.

## Author contributions

HH and JK designed the manuscript. ZW, LS, JWZ, SL, YXZ, RW, YCZ, JHZ, ZZ, JD, and LC performed the literature review. ZW, JHZ, and ZZ drafted the manuscript. ZW, HH, KH, and JK revised the manuscript. All authors contributed to the article and approved the submitted version.
